# Association between apolipoprotein gene polymorphisms and hyperlipidemia: a meta-analysis

**DOI:** 10.1186/s12863-021-00968-1

**Published:** 2021-04-09

**Authors:** Xiao-Ning Zhao, Quan Sun, You-Qin Cao, Xiao Ran, Yu Cao

**Affiliations:** 1 School of Public Health, the Key Laboratory of Environmental Pollution Monitoring and Disease Control, Ministry of Education, Guizhou Medical University, Guiyang, 550025 China; 2 School of Public Health, Hebei Medical University, Shijiazhuang, 050017 China; 3 School of Health, Guizhou Medical University, 550025 Guiyang, China

**Keywords:** Apolipoprotein, APO, Gene polymorphism, Hyperlipidemia, Meta-analysis

## Abstract

**Background:**

Hyperlipidemia plays an important role in the etiology of cardio-cerebrovascular disease. Over recent years, a number of studies have explored the impact of apolipoprotein genetic polymorphisms in hyperlipidemia, but considerable differences and uncertainty have been found in their association with different populations from different regions.

**Results:**

A total of 59 articles were included, containing in total 13,843 hyperlipidemia patients in the case group and 15,398 healthy controls in the control group. Meta-analysis of the data indicated that APOA5–1131 T > C, APOA1 -75 bp, APOB XbaI, and APOE gene polymorphisms were significantly associated with hyperlipidemia, with OR values of 1.996, 1.228, 1.444, and 1.710, respectively. All *P*-values were less than 0.05.

**Conclusions:**

Meta-analysis of the data indicated that the C allele of APOA5 1131 T > C, the A allele at APOA1-75 bp, the APOB XbaI T allele, and the ε2 and ε4 allele of APOE were each a risk factor for susceptibility for hyperlipidemia.

## Background

Cardio-cerebrovascular disease is the leading cause of increased human mortality, globally [1]. Recently, studies have shown that the fatality rate from cardio-cerebrovascular disease accounts for approximately 30% of the total global death toll [2]. Hyperlipidemia is a chronic non-communicable disease caused by an imbalance in the structure of plasma lipids caused by a fat metabolism disorder [3]. It is the primary risk factor for atherosclerosis and the pathological basis for cardio-cerebrovascular disease [4]. In addition, a large number of manuscripts have demonstrated that hyperlipidemia is a pathogenic factor of digestive and urinary diseases such as diabetes, hepatopathy, and pancreatitis*.* Hyperlipidemia can be categorized as hypercholesteremia, hypertriglyceridemia, mixed hyperlipidemia, and low-density lipoproteinemia, etc. Medical research has established that the mechanism of hyperlipidemia is not only determined by environmental factors, such as long-term consumption of large quantities of saturated fatty acids, cholesterol, and sugar, it is also influenced by genetic factors at gene loci. There are multiple academic reports that apolipoprotein (APO) gene mutations are closely related to disorders of blood lipid metabolism [5]. APO is an important component of lipoprotein. So far, more than 20 forms of APO have been identified, including APOA, APOB, APOC, APOD, APOE, APOH, APOM, etc. [6]

Single nucleotide polymorphisms (SNPs) are changes to a single nucleic acid in a protein caused by the insertion, deletion, or substitution of a single nucleotide base in the gene sequence. Of the existing apolipoprotein candidate genes, researchers have correlated APOA1, APOA5, APOB, and APOE gene polymorphisms with hyperlipidemia. APOA1 and APOA5 genes are located in the long arm region of chromosome 11. APOA1 is located in the APOA1-C3-A4 gene cluster, the principal site controlling the expression of lipids and lipoproteins [7]. APOA5 is located downstream of APOA4, and its distance from the APOA1/C3/A4 gene cluster is approximately 30 kb. The APOA5 gene is most commonly altered at -1131 T > C, this polymorphism being closely associated with a number of diseases, such as hypertriglyceridemia and coronary heart disease [8]. The APOB gene is located in the short arm of chromosome 2 and contains 29 exons and 28 introns. The cleavage sites MspI and XbaI are located within exon 26 of the APOB gene. The EcoRI cleavage site is located within exon 29 [9]. A number of studies have clearly indicated that the APOB gene affects lipid metabolism to a certain extent. The APOE gene is located on chromosome 19 with a polymorphic gene structure. The isomers are encoded by the three alleles ε2, ε3, and ε4 [10], forming 6 genotypes E2/2, E3/3, E4/4, E2/3, E2/4, and E3/4, of which E3/3 is the most common within the population.

Over recent years, there have been multiple studies that have explored the correlation between genetic polymorphism and hyperlipidemia for the apolipoprotein gene loci described above, but there are great differences and uncertainties in different populations from different regions. Therefore, in the present review, we systematically searched the literature and reviewed case-control studies of hyperlipidemia. A meta-analysis was conducted to explore the relationship between APOA (A1-75bp, A1 + 83 bp, A5–1131T>C), APOB (MspI, XbaI, EcorI), and APOE with hyperlipidemia so that an evidence-base can be provided for the prevention and control of hyperlipidemia.

## Results

### Study characteristics

**Fig. 1:**
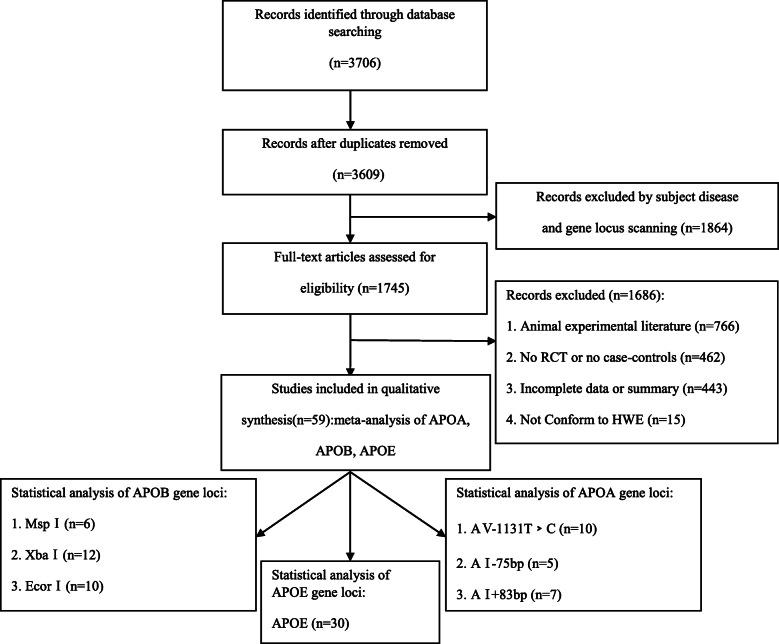
Flow diagram of the meta-analysis

A total of 3706 articles were identified in the Chinese and English databases, of which 59 articles were finally selected, including 22 that analyzed APOA, 28 APOB, and 30 APOE. Three sites in the APOA gene were studied: A5–1131T > C was studied in 10 case-control studies that included 1211 cases and 1495 controls; A1-75bp was studied in 5 case-control studies that included 1284 cases and 1312 controls; and A1 + 83 bp was studied in 7 case-control studies that included 1452 cases and 1620 controls. The APOB gene was investigated at three sites: MspI was studied in 6 case-control studies that included a hyperlipidemia group, with 1155 cases and 1043 controls; XbaI was studied in 12 case-control studies that included 1900 cases and 1836 controls; and EcorI was studied in 10 case-control studies that included 1633 cases and 1686 controls. The APOE gene is co-coded by the three alleles, ε2, ε3, and ε4, for which 30 case control studies were studied that included 5208 cases in the hyperlipidemia group and 6406 cases in the control group. The NOS score of no study included in the review was less than 7. The comparison between case and control groups was highly credible. The specific process for literature retrieval is displayed in Fig. 1.

### Meta-analysis of APOA5–1131 T > C (rs662799)

**Fig. 2:**
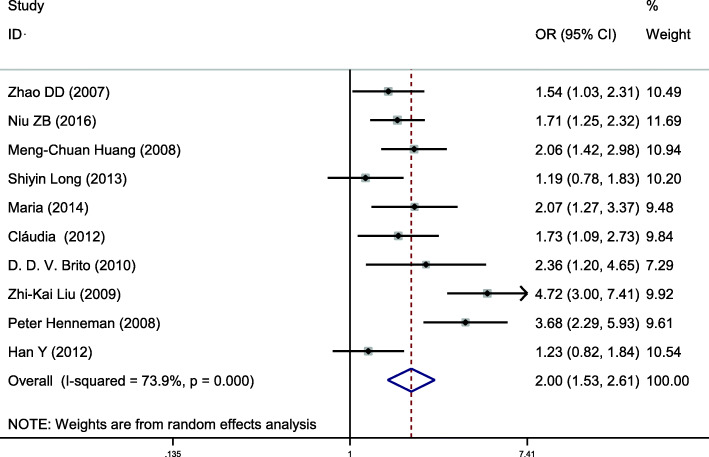
Pooled calculated OR for the association between the APOA5–1131 T > C allele and hyperlipidemia

**Fig. 3:**
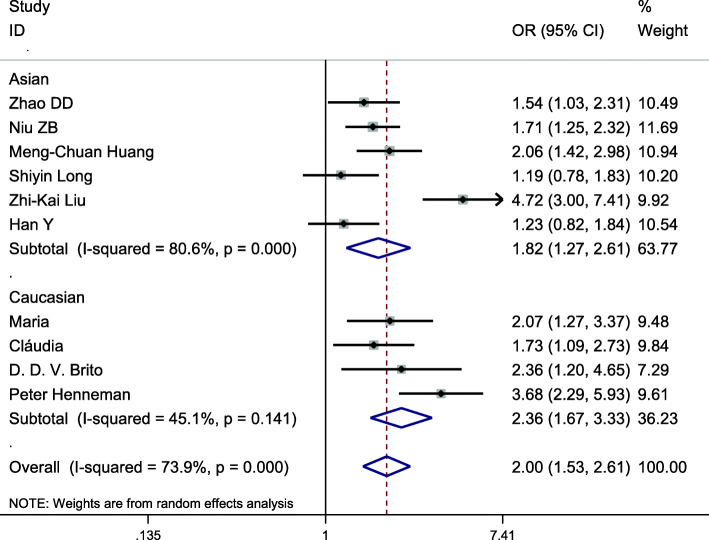
Subgroup analysis by ethnicity for the association between the APOA5–1131 T > C allele and the risk of hyperlipidemia

This gene locus was included in 10 case-control studies, involving a total of 2706 subjects, including 1211 in the hyperlipidemia group and 1496 in the control group. The baseline data and quality evaluation of each study are displayed in Table 1. Analysis of the relationship between C vs T alleles and hyperlipidemia (allele model) revealed substantial heterogeneity (*I*^*2*^ = 73.9%, *P* < 0.001), so a random-effects model was used to analyze the combined effects. Individuals with the C allele had a higher risk of hyperlipidemia than those with the T allele, a difference that was statistically significant (*OR* = 1.996, *95% CI* = 1.529–2.606, *P* < 0.001) (Fig. 2). Other gene models at this site displayed consistent results (Table 2). Subgroup analysis by ethnicity demonstrated an increased risk of hyperlipidemia among Asians (*OR* = 1.818; *95% CI* = 1.268–2.607, *P* = 0.001) and Caucasians (*OR* = 2.355; *95% CI* = 1.665 ~ 3.331, *P* < 0.001) that had the C allele, using the allele model. Other gene models at this site displayed results that were consistent with this (Table 3, Fig. 3). Therefore, the single nucleotide polymorphism APOA5–1131 T > C was associated with hyperlipidemia, the C allele posing a risk factor for susceptibility to hyperlipidemia.

**Table 1:** Main characteristics of the studies of APOA included in the review

SNP	First author	Year	Area	Sample size		Age (y)		Source of control	Genotyping method	Cases			Controls			NOS	HWE	
				Case	Control	Case	Control			TT/GG/CC	CT/GA/CT	CC/AA/TT	TT/GG/CC	CT/GA/CT	CC/AA/TT		*χ*^*2*^	*P*
APOA5–1131 T>C	Zhao DD [11]	2007	Beijing, China	172	80	NR	NR	HB	PCR-RFLP	63	86	23	39	36	5	7	0.77	0.37
	Niu ZB [12]	2016	Shanghai, China	156	262	NR	NR	PB	MALDI-TOF	68	68	20	153	94	15	9	0.01	0.91
	Huang M [13]	2008	Taiwan, China	76	240	59.57 ± 10.2	60.98 ± 13.58	PB	PCR-RFLP	15	41	20	99	111	30	8	0.02	0.9
	Long SY [14]	2013	Hunan, China	95	102	61 ± 12	62 ± 12	HB	PCR-RFLP	46	36	13	50	45	7	7	0.54	0.46
	Maria [15]	2014	Napoli, Italian	165	142	47.5 ± 12.2	43.9 ± 9.6	HB	TaqMan	111	49	5	117	23	2	7	0.49	0.48
	Cláudia [16]	2012	Minas Gerais, Brazil	108	107	48.4 ± 6.8	46.7 ± 6.6	PB	PCR-RFLP	52	52	4	71	33	3	7	0.13	72
	Brito [17]	2010	Belo Horizonte, Brazil	53	77	10.4 ± 2.7	11.2 ± 3.4	HB	PCR-RFLP	34	14	5	62	13	2	6	1.52	0.22
	ZK Liu [18]	2009	Hongkong, China	56	176	49.6 ± 12.3	50.1 ± 9.4	HB	PCR	9	27	20	101	61	11	7	0.19	0.66
	Peter H [19]	2008	Netherlands	254	240	NR	NR	HB	PCR	142	72	7	172	22	1	6	0.11	0.75
	Han Y [8]	2012	Hunan, China	109	117	60.3 ± 12.1	62.9 ± 12.0	HB	PCR-RFLP	52	43	14	59	50	8	7	0.36	0.55
APOA1-75 bp	Huang G [20]	2011	Xinjiang, China	275	252	47.7 ± 7.9	48.23 ± 7.6	HB	PCR-RFLP	135	102	38	136	95	21	8	0.57	0.49
	Feng DW [7]	2016	Xinjiang, China	365	370	46.8 ± 15.9	45.21 ± 16.4	PB	PCR	248	104	13	280	83	7	9	0.09	0.77
	Feng DW [7]	2016	Xinjiang, China	345	391	43.9 ± 14.3	41.5 ± 13.3	PB	PCR	250	87	7	299	86	5	9	0.18	0.67
	Chi YH [21]	2012	Xinjiang,China	200	200	58.5 ± 11.8	58.3 ± 11.5	PB	PCR-RFLP	116	82	2	124	73	5	7	2.31	1.29
	Bora K [2]	2017	Assam, India	100	100	43.1 ± 11.6	43.0 ± 11.6	PB	PCR-RFLP	62	35	3	60	33	7	8	0.68	0.41
APOA1+83 bp	Xie YJ [22]	2011	Xinjiang, China	150	150	56.8 ± 10.8	58.1 ± 10.5	HB	PCR-RFLP	126	24	0	130	20	0	7	0.77	0.38
	Ou HJ [5]	2015	Xinjiang, China	241	246	49.1 ± 0.7	48.3 ± 0.8	HB	MALDI-TOF	160	80	1	171	73	2	7	3.78	0.05
	Feng DW [7]	2016	Xinjiang, China	365	370	46.8 ± 15.9	45.2 ± 16.4	PB	PCR	317	48	0	304	63	3	9	0.02	0.89
	Feng DW [7]	2016	Xinjiang,China	345	391	43.91 ± 14.27	41.51 ± 13.28	PB	PCR	299	44	1	330	57	3	9	0.1	0.76
	Zhu H [23]	2001	Sichuan, China	134	255	54.7 ± 12.6	51.7 ± 10.9	PB	PCR	123	11	0	238	17	0	7	0.3	0.58
	Jia LQ [24]	2005	Sichuan, China	118	109	58.1 ± 8.9	54.5 ± 9.6	NR	PCR	105	13	0	99	10	0	6	0.25	0.62
	Bora K [2]	2017	Assam, India	100	100	43.12 ± 11.64	42.95 ± 11.60	PB	PCR-RFLP	89	11	0	87	13	0	8	0.48	0.49

*SNP* single nucleotide polymorphism, *PB* population-based; HB: hospital-based, *HWE* Hardy-Weinberg equilibrium, *NR* not reported

**Table 2:** Summary of the meta-analysis of the association of APOA gene polymorphisms with hyperlipidemia

SNP	Analysis model	Genotype model	Heterogeneity(*I*^*2*^*/P*)	OR (95%*CI*)	*P*	Publication bias *P*
APOA5–1131 T>C	A	C vs T	73.9%/ < 0.001	1.996(1.529 ~ 2.606)	< 0.001	0.353
	D	TC + CC vs TT	71.2%/ < 0.001	2.179(1.565 ~ 3.035)	< 0.001	0.258
	R	CC vs TC + TT	5.5%/ 0.390	2.790(2.055 ~ 3.789)	< 0.001	0.991
	C	CC vs TT	45.7%/ 0.056	3.604(2.589 ~ 5.017)	< 0.001	0.899
		TC vs TT	67.2%/ 0.001	1.932(1.395 ~ 2.674)	< 0.001	0.465
APOA1-75 bp	A	A vs G	1.2%/ 0.400	1.228(1.067 ~ 1.413)	0.004	0.086
	D	AA+GA vs GG	0.0%/ 0.704	1.246(1.056 ~ 1.471)	0.009	0.067
	R	AA vs GA + GG	15.9%/ 0.313	1.458(0.976 ~ 2.180)	0.066	0.086
	C	AA vs GG	17.4%/ 0.304	1.520(1.008 ~ 2.291)	0.046	0.086
		GA vs GG	0.0%/ 0.828	1.212(1.020 ~ 1.439)	0.029	0.221
APOA1 + 83 bp	A	T vs C	0.0%/ 0.472	0.928(0.771 ~ 1.116)	0.425	0.440
	D	TT + TC vs CC	0.0%/ 0.478	0.950(0.780 ~ 1.157)	0.607	0.371
	R	TT vs TC + CC	0.0%/ 0.799	0.310(0.076 ~ 1.271)	0.104	0.315
	C	TT vs CC	0.0%/ 0.775	0.308(0.075 ~ 1.259)	0.101	0.346
		TC vs CC	0.0%/ 0.607	0.967(0.793 ~ 1.180)	0.740	0.466

*A* allelic model; *D* dominant model; *R* recessive model; *C* codominant model; Publication bias *P*: using Begg’s or Egger’s tests

**Table 3:** Subgroup analysis by ethnicity of the APOA5–1131 T>C polymorphism on susceptibility to hyperlipidemia

Ethnicity	Analysis model	Genotype model	OR (95%*CI*)	*P*
Asian	A	C vs T	1.818(1.268 ~ 2.607)	0.001
	D	TC + CC vs TT	1.943(1.211 ~ 3.117)	0.006
	R	CC vs TC + TT	2.794(2.011 ~ 3.883)	< 0.001
	C	CC vs TT	3.785(1.997 ~ 7.173)	< 0.001
		TC vs TT	1.622(1.060 ~ 2.482)	0.026
Caucasian	A	C vs T	2.355(1.665 ~ 3.331)	< 0.001
	D	TC + CC vs TT	1.943(1.918 ~ 3.749)	< 0.001
	R	CC vs TC + TT	2.790(2.055 ~ 3.789)	0.016
	C	CC vs TT	3.282(1.392 ~ 7.739)	0.007
		TC vs TT	2.600(1.873 ~ 3.609)	< 0.001

*A* allelic model; *D* dominant model; *R* recessive model; *C* codominant model

### Meta-analysis of APOA1-75 bp (rs670)

**Fig. 4:**
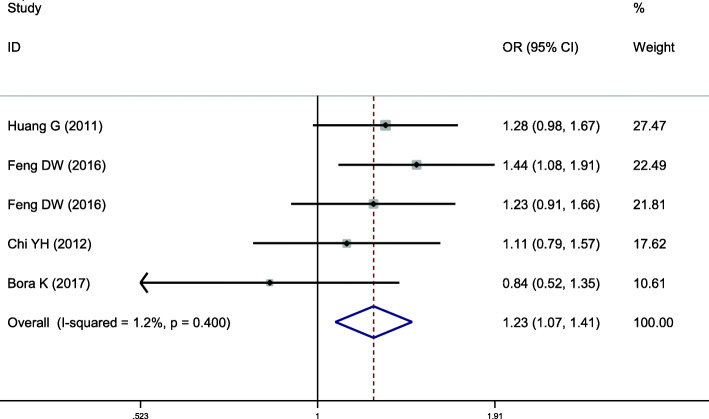
Pooled calculated OR for the association between the APOA1-75 bp allele and hyperlipidemia

This location on APOA was included in 5 case-control studies, involving a total of 2596 subjects, of which 1284 were in the hyperlipidemia group and 1312 in the control group. Baseline data and quality evaluation are displayed in Table 1. There was no significant heterogeneity in the relationship between A vs G alleles and hyperlipidemia (allele model) (*I*^*2*^ = 1.2%, *P* = 0.400), and so a fixed-effects model was used to combine the effects. Individuals with the A allele had a higher risk of hyperlipidemia than those with the G allele, a difference that was statistically significant (*OR* = 1.228, *95% CI* = 1.067–1.413, *P* = 0.004) (Fig. 4). The recessive model of this locus indicated that the difference was not statistically significant (*P* = 0.066). Other gene models at this site were consistent with this result, suggesting that the single nucleotide polymorphism APOA1-75 bp is associated with hyperlipidemia, the A allele being a risk factor for susceptibility to hyperlipidemia (Table 2).

### Meta-analysis of APOA1 + 83 bp (rs5069)

This site was included in 7 case-control studies, involving a total of 3072 subjects, including 1452 in the hyperlipidemia group and 1620 in the control group. The baseline data and quality evaluation of each study are shown in Table 1. Analysis of the relationship between A vs G alleles and hyperlipidemia (allele model) indicated that there was no significant heterogeneity (*I*^*2*^ = 0.0%, *P* = 0.472). Therefore, a fixed-effects model was selected to analyze the pooled effect. There was no significant difference in risk in individuals that carried the T allele compared with C (*OR* = 0.928, *95% CI* = 0.771–1.116, *P* = 0.425). The *P*-values of other gene models at this locus were all higher than 0.05, suggesting that there was no significant difference. Thus, an association between APOA1 + 83 bp gene polymorphism and susceptibility to hyperlipidemia can be considered not to exist (Table 2).

### Meta-analysis of APOB MspI (rs1801701)

This gene locus was included in 6 case-control studies, involving a total of 2198 subjects, including 1155 in the hyperlipidemia group and 1043 in the control group. Baseline data and quality evaluation are shown in Table 4. Analysis of the association between M- vs M+ alleles and hyperlipidemia (allele model) indicated no heterogeneity (*I*^*2*^ = 0.0%, *P* = 0.731), and do a fixed-effects model was selected to analyze the pooled effects. No significant difference in risk was found in individuals carrying the M- compared with the M+ allele (*OR* = 0.892, *95% CI* = 0.756–1.053, *P* = 0.178). The *P-*values of other gene models at this site were also greater than 0.05, indicating that there was no significant difference. Thus, no association between genetic polymorphism of APOB MspI and risk of hyperlipidemia was found (Table 5).

**Table 4:** Principal characteristics of the studies of APOB included in the review

SNP	First author	Year	Area	Sample size		Age (y)		Source of control	Genotyping method	Cases				Controls			NOS	HWE	
				Case	Control	Case	Control			M-M−/TT/ AA	M + M−/CT/ AG	M + M+ /CC/ GG		M-M−/TT/ AA	M + M−/CT/ AG	M + M+ /CC/ GG		*χ*^2^	*P*
APOB Msp	Cao WJ [25]	2009	Xinjiang, China	100	90	46 ± 11	44 ± 11	HB	PCR-RFLP	0	4	95		0	3	87	6	0.03	0.87
	Chi YH [26]	2012	Xinjiang, China	247	221	48.7 ± 7.7	47.3 ± 6.2	HB	PCR-RFLP	9	70	168		6	67	148	7	0.24	0.63
	Huang G [20]	2011	Xinjiang, China	275	252	47.7 ± 7.9	48.2 ± 7.6	HB	PCR-RFLP	25	68	182		22	69	161	8	3.43	0.06
	Jin YN [27]	2015	Chongqing,China	157	180	48.1 ± 3.8	49.1 ± 4.2	HB	DNA chips	0	26	131		0	35	145	7	2.09	0.15
	Chi YH [21]	2012	Xinjiang, China	200	200	58.5 ± 11.8	58.3 ± 11.5	PB	PCR-RFLP	6	66	128		12	64	124	7	0.91	0.34
	Selma [28]	2000	Sao Paulo, Brazil	177	100	58	44	HB	PCR	2	25	150		1	24	75	6	0.37	0.54
APOB XbaI	Qian J [29]	2010	Yunnan, China	91	76	46.9 ± 11.4	47.5 ± 8.1	HB	DNA chips	0	7	84		1	11	64	7	0.42	0.51
	Feng JS [30]	1997	Guangdong, China	108	128	40–70		HB	DNA probe	0	8	100		0	11	117	6	0.26	0.61
	Ma ZZ [31]	2012	Guangdong, China	250	250	45.50 ± 13.20		PB	PCR-RFLP	0	52	198		0	28	222	8	0.88	0.35
	Chi YH [26]	2012	Xinjiang, China	247	221	48.7 ± 7.7	47.3 ± 6.2	HB	PCR-RFLP	4	54	189		3	41	177	7	0.13	0.72
	Xie YJ [22]	2011	Xinjiang, China	150	150	56.8 ± 10.8	58.1 ± 10.5	HB	PCR-RFLP	2	29	119		0	12	138	7	0.26	0.61
	Jin YN [27]	2015	Chongqing,China	157	180	48.1 ± 3.8	49.1 ± 4.2	HB	DNA chips	0	28	129		0	35	145	7	2.09	0.15
	Zhang PZ [32]	2015	Beijing, China	100	100	60.0 ± 5.0		HB	PCR	0	20	80		0	5	95	8	0.07	0.8
	Ou HJ [5]	2015	Xinjiang, China	241	246	49.1 ± 0.7	48.3 ± 0.8	HB	MALDI-TOF	0	19	222		0	32	214	7	1.19	0.28
	Selma [28]	2000	Sao Paulo, Brazil	177	100	58	44	HB	PCR	30	94	53		13	55	32	6	1.99	0.16
	Philippa [33]	1987	London, U.K.	133	62	NR		HB	PCR-RFLP	43	59	31		12	38	12	6	3.16	0.08
	Gong LG [34]	2003	Liaoning, China	115	150	54.2 ± 11.7	52.5 ± 13.1	HB	PCR-RFLP	1	29	85		0	12	138	6	0.26	0.61
	CHOONG [35]	1999	Singapore	131	173	NR		HB	PCR-RFLP	0	25	106		0	21	152	6	0.72	0.4
APOB EcorI	Qian J [29]	2010	Yunnan, China	91	76	46.9 ± 11.4	47.5 ± 8.06	HB	DNA chips	0	13	78		0	3	73	7	0.03	0.86
	Ma ZZ [31]	2012	Guangdong, China	250	250	45.5 ± 13.2		PB	PCR-RFLP	0	41	209		0	28	222	8	0.88	0.35
	Huang G [20]	2011	Xinjiang, China	275	252	47.7 ± 7.9	48.2 ± 7.6	HB	PCR-RFLP	12	73	190		10	77	165	8	0.07	0.79
	Xie YJ [22]	2011	Xinjiang, China	150	150	56.8 ± 10.8	58.1 ± 10.5	HB	PCR-RFLP	1	55	94		0	19	131	7	0.69	0.41
	Jin YN [27]	2015	Chongqing,China	157	180	48.1 ± 3.8	49.11 ± 4.2	HB	DNA chips	0	12	145		0	20	160	7	0.62	0.43
	Zhang PZ [32]	2015	Beijing, China	100	120	60.0 ± 5.0		HB	PCR	1	19	80		1	11	108	8	1.33	0.25
	Ou HJ [5]	2015	Xinjiang, China	241	246	49.1 ± 0.7	48.3 ± 0.8	HB	MALDI-TOF	1	29	211		0	22	224	7	0.54	0.46
	Chi YH [21]	2012	Xinjiang, China	200	200	58.5 ± 11.8	58.3 ± 11.5	PB	PCR-RFLP	6	52	142		6	56	138	7	0.01	0.91
	CHOONG [35]	1999	Singapore	131	173	NR		HB	PCR-RFLP	0	9	122		0	16	157	6	0.41	0.52
	Timirci O [36]	2010	Capa-Istanbul, Turkey	38	39	11.5 ± 3.6	11.4 ± 3.2	HB	PCR	0	4	34		0	4	35	7	0.11	0.74

*SNP* single nucleotide polymorphism, *PB* population-based; HB: hospital-based, *HWE* Hardy-Weinberg equilibrium, *NR* not reported

**Table 5:** Summary of the results of the meta-analysis of the association of APOB gene polymorphisms and hyperlipidemia

SNP	Analysis model	Genotype model	Heterogeneity(*I*^*2*^*/P*)	OR(95%*CI*)	*P*	Publication bias *P*
APOB MspI	A	M- vs M+	0.0%/ 0.731	0.892(0.756 ~ 1.053)	0.178	0.452
	D	M-M−/M + M- Vs M + M+	0.0%/0.716	0.868(0.716 ~ 1.053)	0.152	0.707
	R	M-M-vs M + M−/M + M+	0.0%/ 0.513	0.932(0.596 ~ 1.456)	0.757	0.908
	C	M-M- vs M + M+	0.0%/ 0.555	0.903(0.574 ~ 1.421)	0.660	0.883
		M + M- vs M + M+	0.0%/ 0.654	0.864(0.705 ~ 1.057)	0.156	0.746
APOB XbaI	A	T vs C	72.4%/ < 0.001	1.444(1.061 ~ 1.966)	0.020	0.732
	D	TT + CT vs CC	73.5%/ < 0.001	1.360(0.943 ~ 1.962)	0.100	0.945
	R	TT vs CT + CC	0.0%/ 0.747	1.613(1.022 ~ 2.545)	0.040	0.707
	C	TT vs CC	0.0%/ 0.774	1.432(0.851 ~ 2.411)	0.017	0.724
		CT vs CC	73.5%/ < 0.001	1.322(0.912 ~ 1.917)	0.140	0.837
APOB EcorI	A	A vs G	70.0%/ < 0.001	1.333(0.942 ~ 1.885)	0.104	0.474
	D	AA+AG Vs GG	72.9%/ < 0.001	1.366(0.924 ~ 2.020)	0.118	0.283
	R	AA vs AG + GG	0.0%/ 0.942	1.183(0.628 ~ 2.229)	0.603	0.221
	C	AA vs GG	0.0%/ 0.886	1.166(0.617 ~ 2.202)	0.637	0.086
		AG vs GG	72.6%/ < 0.001	1.356(0.913 ~ 2.015)	0.131	0.371

*A* allelic model; *D* dominant model; *R* recessive model; *C* codominant model; Publication bias *P*: using Begg’s or Egger’s tests

### Meta-analysis of APOB XbaI (rs693)

**Fig. 5:**
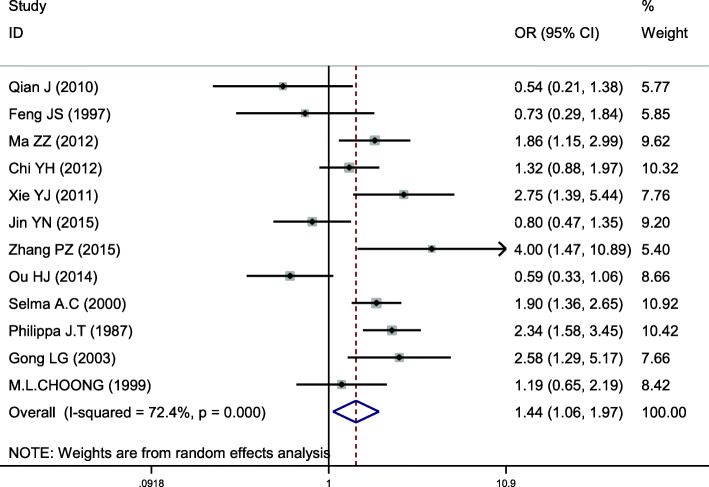
Pooled calculated OR for the association between the APOB XbaI allele and hyperlipidemia

**Fig. 6:**
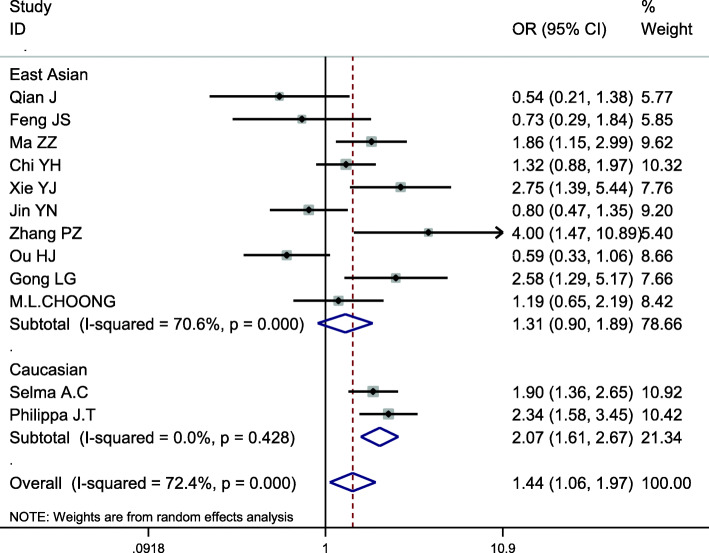
Subgroup analysis by ethnicity for the association between the APOB XbaI allele and the risk of hyperlipidemia

This site was included in 12 case-control studies, involving a total of 3736 subjects, including 1900 in the hyperlipidemia group and 1836 in the control group. Baseline data and quality evaluation are shown in Table 4. Analysis of the association between T vs C alleles and hyperlipidemia (allele model) indicated substantial heterogeneity (*I*^*2*^ = 72.4%,*P* < 0.001) and so a random-effects model was used to analyze the pooled effects. The risk of hyperlipidemia in the T allele population was higher than that with the C allele, the difference of which was statistically significant (*OR* = 1.444, *95% CI* = 1.061–1.966, *P* = 0.020) (Fig. 5). There was no significant difference between the dominant and codominant models of this locus, with *P*-values of 0.100 and 0.140, respectively. The results of other gene models were consistent with those of the allele model (Table 5). Subgroup analysis by ethnicity displayed an increased risk of hyperlipidemia among Caucasians that carried the T allele when analyzed with the allele model, a difference that was statistically significant (*OR* = 2.074; *95% CI* = 1.611–2.672, *P* < 0.001). However, no significant association was found in other gene models. We found that there was no significant association with risk of hyperlipidemia risk in Asians carrying the T allele using the allele model (*OR* = 1.305; *95% CI* = 0.902–1.888, *P* = 0.158), other gene models displaying results consistent with those of the allele model (Table 6, Fig. 6). Therefore, an association between APOB XbaI gene single nucleotide polymorphism and hyperlipidemia in Asians was not considered to exist. However, the T allele at this locus could be considered a risk factor for hyperlipidemia in Caucasians.

**Table 6:** Subgroup analysis by ethnicity of the APOB XbaI polymorphism on susceptibility to hyperlipidemia

Ethnicity	Analysis model	Genotype model	OR(95%CI)	P
Asian	A	T vs C	1.305(0.902 ~ 1.888)	0.158
	D	TT + CT vs CC	1.470(0.953 ~ 2.267)	0.081
	R	TT vs CT + CC	1.476(0.507 ~ 4.300)	0.475
	C	TT vs CC	1.569(0.542 ~ 4.541)	0.406
		CT vs CC	1.466(0.960 ~ 2.238)	0.077
Caucasian	A	T vs C	2.075(1.611 ~ 2.672)	< 0.001
	D	TT + CT vs CC	0.985(0.640 ~ 1.518)	0.947
	R	TT vs CT + CC	1.644(0.993 ~ 2.723)	0.053
	C	TT vs CC	1.391(0.765 ~ 2.530)	0.280
		CT vs CC	0.848(0.509 ~ 1.412)	0.526

*A* allelic model; *D* dominant model; *R* recessive model; *C* codominant model

### Meta-analysis of APOB EcorI (rs1042031)

This site was included in 10 case-control studies, involving a total of 3319 subjects, including 1633 in the hyperlipidemia group and 1686 in the control group. Baseline data and quality evaluation are shown in Table 4. Analysis of the association between A vs G alleles and hyperlipidemia (allele model) indicated heterogeneity (*I*^*2*^ = 70.0%, *P* < 0.001), so the pooled effects were analyzed using a random-effects model. There was no significant difference in risk in individuals carrying the A or G alleles (*OR* = 1.333, *95% CI* = 0.942–1.885, *P* = 0.104). The results of other gene models at this site were consistent with this conclusion, and so no association between the genetic polymorphism of APOB Ecor I and susceptibility to hyperlipidemia (Table 5) can be considered to exist.

### Meta-analysis of APOE

**Fig. 7:**
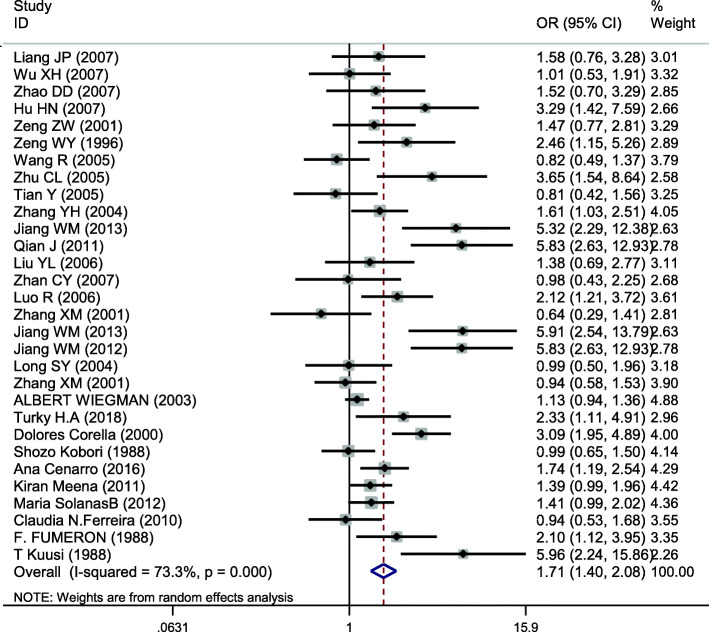
Pooled calculated OR for the association between the APOE allele and hyperlipidemia

**Fig. 8:**
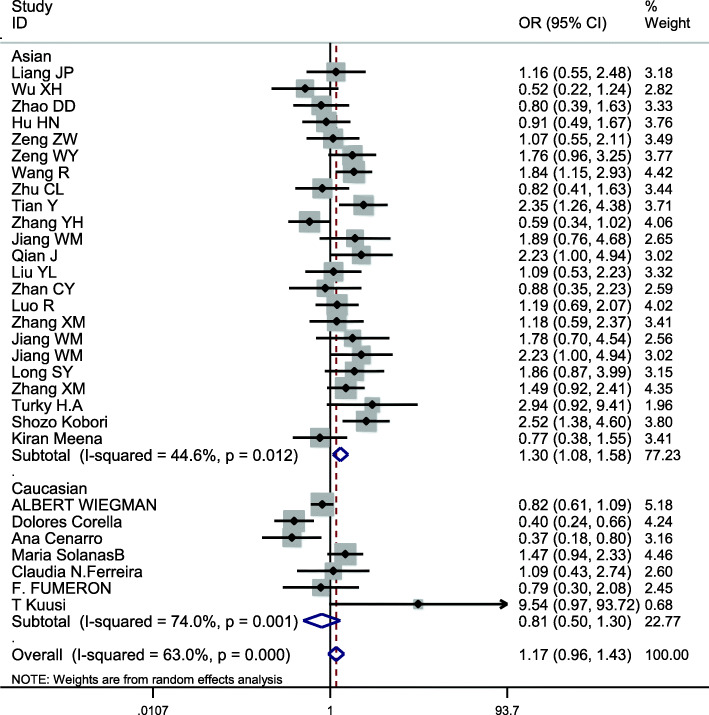
Subgroup analysis by ethnicity for the association between the APOE ε2 and ε3 alleles and the risk of hyperlipidemia

**Fig. 9:**
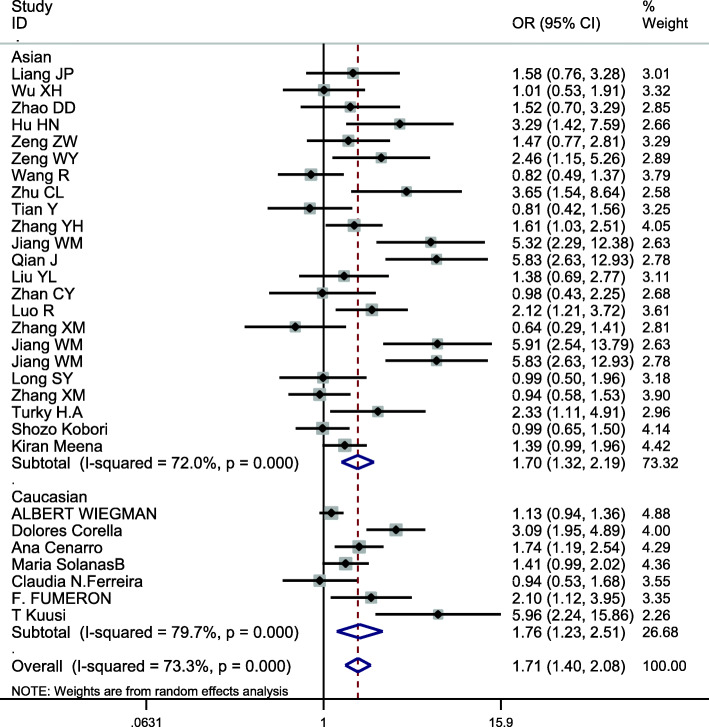
Subgroup analysis by ethnicity for the association between the APOE ε3 and ε4 alleles and the risk of hyperlipidemia

This site was included in 30 case-control studies, involving a total of 11,614 subjects, including 5208 in the hyperlipidemia group and 6406 in the control group. The baseline data and quality evaluation of the various studies are displayed in Table 7. The APOE ε3 allele was used as a reference to analyze the relationship between alleles and hyperlipidemia. Analysis of the data for ε2 (*I*^*2*^ = 63.0%, *P* < 0.001) and ε4 (*I*^*2*^ = 73.3%, *P* < 0.001) indicate that heterogeneity was present and so the pooled effects were analyzed using a random-effects model. The difference in risk between individuals with the ε2 and ε3 allele was not statistically significant (*OR* = 1.167, *95% CI* = 0.955–1.426, *P* = 0.131). The risk of hyperlipidemia in individuals with the ε4 allele was higher than in those with the ε3 allele, a difference that was statistically significant (*OR* = 1.710, *95% CI* = 1.405–2.083, *P* < 0.001) (Fig. 7). Because of heterogeneity, subgroup analysis by ethnicity was conducted, the results using the allele model demonstrating a risk of hyperlipidemia was different for Asians (*OR* = 1.304; *95% CI* = 1.075–1.582, *P* = 0.007) for those with ε2 compared with the ε3 allele, but the association was not significant for Caucasians (*OR* = 0.807; *95% CI* = 0.502–1.297, *P* = 0.376) (Fig. 8). There were significant differences in risk of hyperlipidemia, which was higher in both Asians (*OR* = 1.704; *95% CI* = 1.325–2.192, *P* < 0.001) and Caucasians (*OR* = 1.759; *95% CI* = 1.231–2.513, *P* = 0.002) with the ε4 allele than those carrying the ε3 allele (Fig. 9).

**Table 7:** Main characteristics of the studies of APOE included in the review

First author	Year	Area	Sample size		Age (y)		Source of control	Genotyping method	Cases							Controls						NOS	HWE	
			Case	Control	Case	Control			E2/E2	E2/E3	E2/E4	E3/E3	E3/E4	E4/E4		E2/E2	E2/E3	E2/E4	E3/E3	E3/E4	E4/E4		*χ*^*2*^	*P*
Liang JP [37]	2008	Beijing,China	210	94	58.48	NR	HB	PCR-RFLP	2	19	2	155	32	0		0	9	1	75	9	0	6	0.94	0.63
Wu XH [38]	2007	Xinjiang,China	100	91	48.7 ± 10.5	43.1 ± 10.8	HB	PCR-RFLP	0	9	0	69	21	1		0	13	2	60	14	2	6	1.79	0.41
Zhao DD [11]	2007	Beijing,China	172	80	NR		HB	PCR-RFLP	1	18	2	124	27	0		0	13	0	58	9	0	7	2.03	0.36
Hu HN [39]	2007	Hubei,China	165	108	60.5 ± 8.3	63.8 ± 6.2	HB	ARMS-PCR	0	26	0	109	27	3		0	20	0	81	7	0	7	2.2	0.33
Zeng ZW [40]	2001	Guangdong,China	163	87	56.4 ± 3.2	58.0 ± 2.4	HB	PCR-RFLP	0	22	5	104	32	0		0	12	2	61	12	0	6	1.82	0.4
Zeng WY [41]	1996	Beijing,China	133	122	41–60		PB	PCR	5	17	4	88	18	1		1	14	2	97	8	0	7	2.87	0.24
Wang R [42]	2005	Sichuan,China	206	250	52	51	HB	PCR-RFLP	0	46	2	135	22	1		2	28	1	182	35	2	7	1.9	0.39
Zhu CL [43]	2005	Hubei,China	113	108	62.5 ± 7.2	63.8 ± 6.2	HB	ARMS-PCR	0	16	0	74	21	2		0	20	0	81	7	0	7	2.2	0.33
Tian Y [44]	2005	Sichuan,China	103	146	56.9 ± 8.5	56.3 ± 9.8	PB	PCR-RFLP	2	23	1	64	12	1		1	15	1	102	27	0	8	2.53	0.28
Zhang YH [45]	2004	Beijing,China	160	328	47.3 ± 13.8	40.1 ± 13.5	PB	PCR-RFLP	0	13	5	114	22	6		0	55	8	225	38	2	7	5.59	0.06
Jiang WM [46]	2013	Jiangsu,China	102	100	48.4 ± 9.7	50.2 ± 15.1	HB	DNA sequencing	1	9	2	64	22	4		0	7	1	86	6	0	7	2.19	0.33
Qian J [47]	2011	Jiangsu,China	212	100	54.6 ± 11.9	50.2 ± 15.1	HB	DNA sequencing	2	21	6	127	47	9		0	7	1	86	6	0	7	2.19	0.33
Liu YL [48]	2006	Shanxi,China	72	95	NR		HB	ARMS-PCR	2	8	3	45	13	1		0	16	3	61	15	0	7	2.66	0.26
Zhan CY [49]	2007	Beijing,China	96	95	60.0 ± 8.3	NR	HB	PCR	0	9	0	75	12	0		0	9	1	75	9	1	7	1.75	0.42
Luo R [50]	2006	Hubei,China	164	156	58.3 ± 7.1	53.1 ± 4.7	HB	PCR-RFLP	1	27	1	101	28	6		1	21	3	116	13	2	6	5.04	0.08
Zhang XM [51]	2001	Sichuan,China	74	230	56.8 ± 12.4	51.3 ± 10.3	PB	PCR-RFLP	0	10	2	56	6	0		2	26	1	165	35	1	7	2.27	0.32
Jiang WM [52]	2013	Jiangsu,China	93	100	56.0 ± 11.85	50.2 ± 15.1	HB	DNA sequencing	1	7	2	57	22	4		0	7	1	86	6	0	6	2.19	0.33
Jiang WM [53]	2012	Jiangsu,China	212	100	54.6 ± 11.85	50.2 ± 15.1	HB	DNA sequencing	2	21	6	127	47	9		0	7	1	86	6	0	6	2.19	0.33
Long SY [54]	2004	Sichuan,China	112	73	58.2 ± 7.9	55.1 ± 9.7	PB	PCR-RFLP	1	21	4	68	17	1		1	8	0	48	16	0	7	3.89	0.14
Zhang XM [55]	2001	Sichuan,China	225	230	53.0 ± 15.5	51.3 ± 10.3	PB	PCR-RFLP	1	37	5	156	23	3		2	26	1	165	35	1	7	2.27	0.32
ALBERT [56]	2003	Amsterdam, Netherlands	450	2018	10.8	NR	HB	PCR	0	50	10	243	135	12		13	261	45	1128	512	59	7	2.83	0.24
Turky H.A [57]	2018	Riyadh, Saudi Arabia	104	100	57.8 ± 9.9	44.0 ± 6.3	HB	TaqMan	1	7	2	74	18	2		0	4	0	85	11	0	8	0.66	0.72
Corella [58]	2000	Valencia, Spain	330	330	38.8 ± 9.1	37.6 ± 8.4	PB	PCR	0	17	5	237	69	2		3	50	1	252	23	1	7	1.28	0.53
Kobori [59]	1988	Kumamoto, Japan	447	188	30–69		HB	SRID	9	49	7	323	47	12		0	12	1	143	30	2	7	0.39	0.82
Cenarro [60]	2016	Zaragoza, Spain	288	220	47.9 ± 11.5	44.8 ± 16.0	HB	RT-PCR	0	9	1	186	72	11		0	19	3	160	34	4	8	2.53	0.28
Kiran [61]	2011	New Delhi, India	219	352	42.0 ± 7.9	35.2 ± 9.6	HB	PCR-RFLP	0	8	4	143	62	2		2	19	3	251	73	4	7	5.48	0.06
SolanasB [62]	2012	Zaragoza, Spain	312	264	48.4 ± 9.7	43.5 ± 16.9	HB	PCR	11	25	5	189	65	8		1	27	4	183	45	4	8	0.46	0.79
N.Ferreira [63]	2010	Minas Gerais, Brasil	109	107	48.4 ± 6.8	46.7 ± 6.6	HB	PCR-RFLP	0	10	0	77	18	4		0	9	0	72	25	1	7	2.26	0.32
FUMERON [64]	1988	Paris, France	59	113	NR		HB	PCR	0	5	1	35	14	4		1	13	1	79	16	3	6	3.96	0.14
T Kuusi [65]	1988	Helsinki, Finland	21	21	45.2 ± 0.8	46.7 ± 1.5	HB	PCR	0	1	3	2	8	7		0	1	0	11	8	1	6	0.44	0.8

*SNP* single nucleotide polymorphism, *PB* population-based, *HB* hospital-based, *HWE* Hardy-Weinberg equilibrium, *NR* not reported, *SRID* single radial immunodiffusion

Correlations in the APOE genotype (E2/E2, E2/E3, E2/E4, E3/E4, E4/E4) and hyperlipidemia were analyzed using the wild type E3/E3 genotype as a reference. The heterogeneity, and OR and 95% CI values of these data are displayed in Table 8. The significance level was adjusted to α′ = α/(k-1) = 0.01. There was a significant difference in risk of hyperlipidemia between carriers of the E2/E4, E3/E4, and E4/E4 genotypes with carriers of the E3/E3 genotype, the *P*-values of which were < 0.01 in each case. To identify the source of significant heterogeneity, we conducted subgroup analysis based on ethnicity. The results demonstrated that there was a significant difference in risk of hyperlipidemia in carriers of all genotypes (E2/E2, E2/E3, E2/E4, E3/E4, E4/E4) compared with carriers of the E3/E3 genotype in Asians, while Caucasians carrying the E3/E4, E4/E4 genotypes were statistically different from those carrying E3/E3 (Table 9). Therefore, APOE gene polymorphisms can be considered to be closely associated with hyperlipidemia. For Asians, either the ε2 or ε4 allele was a risk factor for hyperlipidemia, while for Caucasians, only the ε4 allele was a risk factor.

**Table 8:** Summary of the meta-analysis of the association of APOE gene polymorphisms with hyperlipidemia

Genotype model	Heterogeneity(*I*^*2*^*/P*)	OR(95%*CI*)	*P*	publication bias *P*
E2/E2	0.0%/0.634	1.746(1.081 ~ 2.819)	0.023	0.131
E2/E3	50.3%/0.001	1.076(0.883 ~ 1.311)	0.467	0.400
E2/E4	0.0%/0.790	1.693(1.227 ~ 2.336)	0.001	0.054
E3/E4	67.8%/< 0.001	1.578(1.276 ~ 1.951)	< 0.001	0.073
E4/E4	2.7%/ 0.424	2.346(1.723 ~ 3.195)	< 0.001	0.851

Publication bias *P*: using Begg’s or Egger’s tests

**Table 9:** Subgroup analysis by ethnicity of APOE gene polymorphisms on susceptibility to hyperlipidemia

Ethnicity	Genotype model	OR(95%CI)	P
Asian	E2/E2	2.062(1.131 ~ 3.761)	0.003
	E2/E3	1.229(1.006 ~ 1.502)	0.009
	E2/E4	1.958(1.283 ~ 2.986)	0.002
	E3/E4	1.579(1.201 ~ 2.077)	0.001
	E4/E4	3.312(2.041 ~ 5.374)	< 0.001
Caucasian	E2/E2	1.248(0.549 ~ 2.841)	0.597
	E2/E3	0.703(0.479 ~ 1.034)	0.073
	E2/E4	1.342(0.805 ~ 2.237)	0.260
	E3/E4	1.612(1.121 ~ 2.317)	0.002
	E4/E4	1.712(1.129 ~ 2.596)	0.002

### Publication bias and sensitivity analysis

**Fig. 10:**
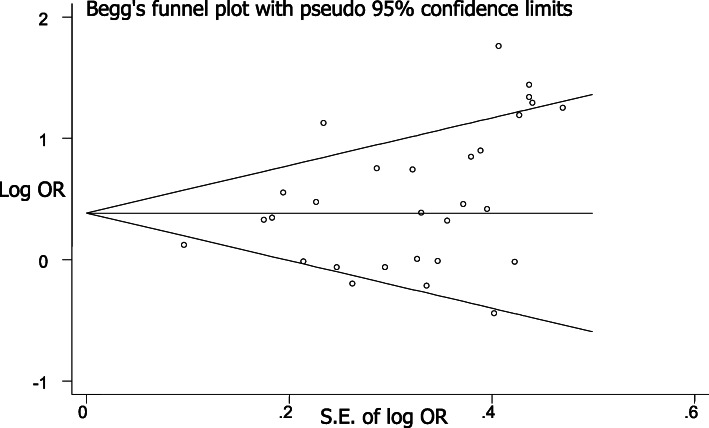
Begg’s funnel plot for the APOE ε4 allele

There was no apparent asymmetry in each Begg’s funnel plot (Fig. 10), indicating that publication bias was slight. In addition, statistical analysis of the symmetry of Begg’s funnel plots using an Egger’s test demonstrated that publication bias for each gene locus displayed *P*-values all > 0.05, indicating that publication bias was apparently not present.

For groups that deviated substantially in the analysis, meta-analysis was performed again after exclusion of the associated manuscripts, and OR and *P*-values re-calculated. Exclusion of the study [18] for APOA5–1131 T > C with the most deviating OR value using the allele model resulted in conclusions similar and consistent with those of the original data (*OR* = 1.800, *95% CI* = 1.454–2.229, *P* < 0.001). The results indicated stability in the APOA1-75 bp and APOA1 + 83 bp allele models, with no literature having excessive deviation.

For the APOB Xba I locus using the allele model, exclusion of the manuscript [32] with the largest deviation in OR value resulted in conclusions of the meta-analysis consistent with the original conclusions (*OR* = 1.365, *95% CI* = 1.001–1.862, *P* = 0.049). Exclusion of the biased literature [36] that studied APOB Ecor I in Caucasians resulted in differences in the meta-analysis that were not statistically significant and consistent with the original conclusions (*OR* = 1.351, *95% CI* = 0.940–1.941, *P* = 0.104). Sensitivity analysis of the allele model of APOB Msp I was performed, the results of which were consistent with the original conclusions (*OR* = 0.926, *95% CI* = 0.779–1.102, *P* = 0.387).

Exclusion of the manuscript [65] with the greatest deviation in data for the ε2 allele of APOE resulted in conclusions for the meta-analysis that the ε2 allele was not associated with hyperlipidemia (*OR* = 1.150, *95% CI* = 0.943–1.402, *P* = 0.167). Correspondingly, exclusion of the literature [65] with the largest deviation for the APOE ε4 allele resulted in conclusions consistent with those originally recorded, following recalculation, and so carrying the ε4 allele can be considered a risk factor for hyperlipidemia (*OR* = 1.657, *95% CI* = 1.365–2.012, *P* < 0.001). To summarize, we conclude that there was no apparent inconsistency in the literature that would contradict our original conclusions, with good reliability.

## Discussion

The present study found that allele C at APOA5–1131 T > C was a risk factor for hyperlipidemia, the A allele at AI-75 bp conferred susceptibility to hyperlipidemia, the T allele at APOB Xba I represents a preliminary pathogenic factor for hyperlipidemia in Caucasians, allele ε4 of the APOE gene is a risk factor for hyperlipidemia, and allele ε2 is a risk factor for hyperlipidemia in Asians.

The APOE gene, located on chromosome 19, contains 4 exons and 3 introns, with 3 isomers, and the functions by of regulating plasma total cholesterol (TC) and lipoprotein metabolism. APOE3 is the most common phenotype. A principal function is to bind low-density lipoprotein receptor (LDL-R) and APOE receptor as the ligand [66]. Compared with APOE3, the ability of APOE4 to bind to its receptor is relatively strong, resulting in the metabolism of chylomicrons (CMs) and very low-density lipoprotein (VLDL) residues to be accelerated and the conversion of VLDL to LDL to increase. Additionally, the rate of liver internalization and catabolism of CM and VLDL residues becomes accelerated, resulting in increased free cholesterol in hepatocytes with feedback that caused a down-regulation of LDL-R on their surface, resulting in a decrease in the metabolic rate of LDL [67]. Furthermore, the low intestinal cholesterol absorption capacity of ε4 carriers also increases, resulting in higher plasma levels of TC and LDL. This is consistent with the conclusion that the ε4 allele is a risk factor for hyperlipidemia in the present review. The study also found that the ε2 allele is harmful for blood lipid levels in the Asian population, but failed to establish the effects on blood lipid levels in the Caucasian population. This may be related to the imbalance of internal composition and the small sample size for Caucasians. Of course, we cannot rule out the possibility of a corresponding biological mechanism to explain why this locus has no harmful effects on Caucasians.

APOB is the principal protein component of LDL and plays a role in transportation of endogenous cholesterol to maintain its balance within the body. The APOB gene is located in region 23–24 of the short arm of human chromosome 2. The APOB gene plays a key role in the production, transport, and removal of LDL and VLDL from plasma and regulates the concentration of plasma cholesterol [68]. The polymorphism of the APOB XbaI restriction site is due to a mutation of nucleotide C → T at position 7673 of the APOB gene cDNA, which changes the codon sequence at position 2488 (ACC → ACT), thus producing an XbaI endonuclease recognition site. The T allele may be related to a reduction in LDL degradation rate mediated by the receptor [9]. A number of studies have also speculated that a single nucleotide polymorphism at this locus is a genetic marker and has linkage disequilibrium with other nearby DNA sequence variants that affect cholesterol levels [69]. Such a molecular mechanism could explain why the T allele is a risk factor for hyperlipidemia in Caucasians. Other studies further confirm our conclusions that this polymorphism of the APOB XbaI gene might increase the risk of cerebral infarction, and that the T allele is such a risk factor [70]. The T allele was associated with lower levels of HDL-C, which may be associated with the incidence of coronary heart disease [71].

The APOA1 gene is located in the terminal region of the long arm of chromosome 11 and consists of 3 introns and 4 exons. APOA1 is the main apolipoprotein to create high-density lipoprotein (HDL), maintaining the stability and integrity of the HDL structure, and promoting the esterification of cholesterol (TC) [72]. The APOA1-75 bp polymorphism not only destroys the endonuclease recognition site but also changes the GGGCCGG sequence which activates transcription. A change in the sequence may also affect the synthesis of APOA1 [73]. This mechanism is consistent with the conclusion that there is an association between the A1-75bp gene single nucleotide polymorphisms and hyperlipidemia. The APOA5 gene, located in 23 regions of the long arm of chromosome 11, has 1889 bps and consists of 4 exons, 2 introns, and 4 silencing molecules. APOA5 can reduce triglyceride (TG) and increase HDL, representing a protective factor for coronary heart disease [74]. Some of the manuscripts also clearly stated that the mutation APOA5–1131 T > C is closely related to increased triglyceride levels [75] and that the CC genotype of this locus was positively correlated with serum TG levels and negatively correlated with APOA5 levels [76].

A meta-analysis can effectively compensate for the lack of statistical efficacy and other problems within a single study. However, although the present review developed a scientifically-based and comprehensive search strategy with strict unified screening criteria, limitations still remain [77]: (1) There were few relevant Chinese and English manuscripts on the acquisition of particular gene loci, such as APOAI and APOB MspI, so the number of case-control studies included in the analysis was small, possibly reducing the effectiveness of the Egger’s and Begg’s tests, in addition to sensitivity analysis; (2) The data included in the review did not involve additional races, which led to heterogeneity. Although ethnic subgroup analysis can identify heterogeneity to some extent, we found that there was a small sample size in Caucasians for APOB XbaI, possibly the reason why the results of the genetic model were not consistent at this locus. (3) It is unknown whether there were statistical differences in sex and age among individuals included in the study; (4) The effects of gene-environmental interactions and genetic linkage disequilibrium were not considered. In the future, we shall include more reliable data in this respect and update the meta-analysis, thereby providing a more reliable evidence base for the prevention and control of hyperlipidemia from the perspective of the apolipoprotein gene.

## Conclusions

In summary, the results of the present meta-analysis revealed that the C allele of APOA5 1131 T > C, the A allele at APOA1-75 bp, the APOB XbaI T allele, and the ε2 and ε4 alleles of APOE may represent genetic risk factors for susceptibility for hyperlipidemia. In addition, we found it is consistent with the present study on the pathological mechanisms of hyperlipidemia. However, there is a need for further large-scale studies, including larger case-control studies and analysis of other loci of the APO genes, to confirm our conclusions and elucidate the influence of gene-environment interactions.

## Methods

### Literature search strategy

The Pubmed, Web of Science, ScienceDirect, the Chinese National Knowledge Infrastructure database, the Chinese Wanfang database, and Database of Chinese science and technology periodicals were searched to identify studies that evaluated the association of APO gene polymorphisms with the risk of hyperlipidemia, where publication date was prior to June 9, 2020. The keywords “apolipoprotein”, “APO”, “hyperlipidemia”, “dyslipidemias”, “hypercholesteremia”, “hypertriglyceridemia”, “mixed hyperlipidemia”, “low density lipoproteinemia”, “APOA”, “APOB”, “APOC”, “APOD”, “APOE”, “APOA5–1131 T > C”, “rs662799”, “APOA1-75 bp”, “rs670”, “APOA1 + 83 bp”, “rs5069”, “APOB MspI”, “rs1801701”, “APOB XbaI”, “rs693”, “APOB EcorI”, “rs1042031”, “gene”, “polymorphism”, and “genetic polymorphism” were searched. The references of all eligible studies were also searched manually in order to find other studies missed during the initial search activity. The analysis followed the guidelines of the Preferred Reporting Items for Systematic Reviews and Meta-Analysis (PRISMA) statement [78].

### Identification of studies for inclusion

The inclusion criteria for the present meta-analysis were as follows: (1) studies that evaluated the association between APO and risk of hyperlipidemia; (2) studies with an appropriate statistical design and selection methods; (3) case-control and RCT studies; (4) diagnostic criteria for dyslipidemia that were clear and uniform [79]; (5) distribution of APO genotypes in controls group were consistent with the Hardy-Weinberg equilibrium (HWE); (6) allele typing methods were accurate; (7) data included in the studies were complete, without omissions. Duplicated data, reviews, abstracts, case reports, animal studies, and studies that did not meet the inclusion criteria were excluded.

### Data extraction

Two reviewers (XNZ and QS) independently conducted literature screening and evaluation. The following information was extracted from each study for inclusion in the review: first author, year of publication, area, age, source of control, sample size of controls and cases, genotyping method, Hardy-Weinberg equilibrium (HWE), and the distribution of genotypes and frequencies of alleles in cases and controls. Any disputes were resolved by discussion with a third investigator.

### Quality evaluation

The quality of the selected case-control studies was evaluated according to the Newcastle-Ottawa Quality Assessment Scale (NOS) [80], of which data with scores 0–3, 4–6 or 7–9 were low, moderate or high-quality, respectively [81].

### Statistical analyses

The included hyperlipidemia data were analyzed by meta-analysis using Stata 11 software. The correlation between apolipoprotein gene polymorphism and hyperlipidemia was expressed by odds ratio (OR) and 95% confidence intervals (CIs). In order to better evaluate the presence of heterogeneity between the studies, an *I*^*2*^ test was also used. Where homogeneity (*I*^*2*^ < 50%) was identified in the meta-analysis, a fixed-effects model was adopted; otherwise, a random-effects model was used to integrate the incorporated data. The data were assessed using Egger’s and Begg’s tests to evaluate publication bias. Sensitivity analysis was conducted by deleting, in turn, the data from individual studies that had large deviations as identified in the results, then recalculating the OR value. All *P*-values were two-sided, with a significance threshold set at α *=* 0.05.

To explore the source of significant heterogeneity, subgroup analysis of race was performed. A total of 7 sites were included, of which 3 sites (APOA5–1131 T > C,APOB XbaI, and APOE) were evaluated by subgroup analysis of ethnicity, 2 sites (APOB MspI, and APOB EcorI) were analyzed by sensitivity analysis, as there was only one published study of different races in the literature that was not suitable for subgroup analysis. Race was not evaluated in 2 sites (APOA1-75 bp, APOA1 + 83 bp) by subgroup analysis due to the fact that the populations studied were the same race, and had no significant heterogeneity.

## Data Availability

All data analysed in this study can be derived from publicly available databases.

## Abbreviations

APO: Apolipoprotein
SNPs: Single nucleotide polymorphisms
HWE: Hardy-Weinberg Equilibrium
NOS: Newcastle-Ottawa Quality Assessment Scale
TC: Total cholesterol
LDL-R: Low-density lipoprotein receptor
CM: Chylomicron
VLDL: Very low-density lipoprotein
HDL: High-density lipoprotein
